# Supplementary material for: Time‑resolved multi-omic analysis of paclitaxel exposure in human iPSC‑derived sensory neurons unveils mechanisms of chemotherapy‑induced peripheral neuropathy
Source: Cell Death Dis. 2026 Feb 10;17(1):211. doi: 10.1038/s41419-026-08445-2 (PMC12921266; doi:10.1038/s41419-026-08445-2)
Supplement: Supplementary file 15 — Dataset 1. [file 41419_2026_8445_MOESM15_ESM.zip › CDD_Open_Data/04_RNA_Seq/Timeline_panel_plots_etc_2023-01-23_1609.zip!/Panel_Plots_etc/Evidence_Plots/24h/evidence_plot_standard contrasts_msigdb_reactome_24h_PTX_vs_DMSO_ID4_M16227_cholesterol.pdf]

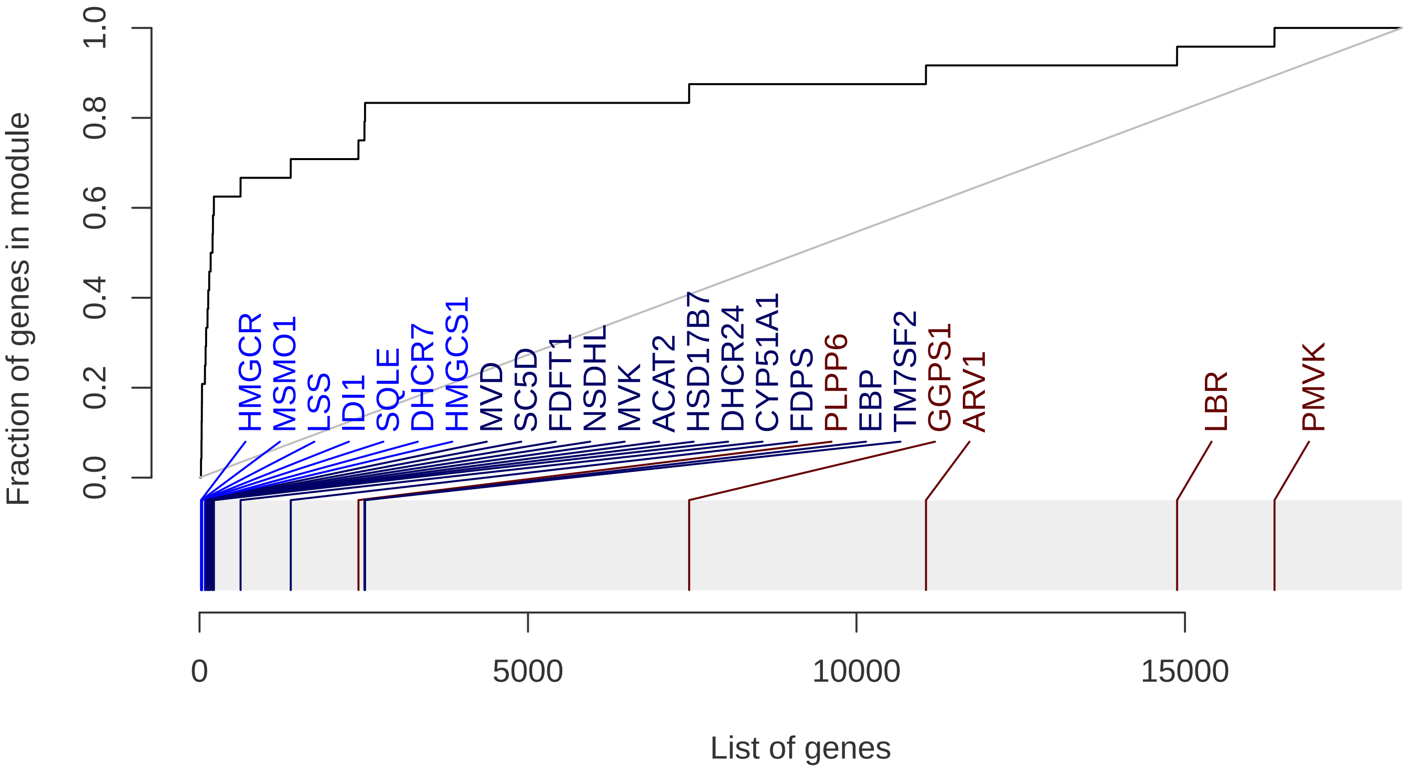

Fraction of genes in module
0.0
0.2
0.4
0.6
0.8
1.0
HMGCR
MSMO1
LSS
IDI1
SQLE
DHCR7
HMGCS1
MVD
SC5D
FDFT1
NSDHL
MVK
ACAT2
HSD17B7
DHCR24
CYP51A1
FDPS
PLPP6
EBP
TM7SF2
GGPS1
ARV1
LBR
PMVK
0
5000
10000
15000
List of genes
